# Lower fitness levels, higher fat-to-lean mass ratios, and lower cardiorespiratory endurance are more likely to affect the body mass index of Saudi children and adolescents

**DOI:** 10.3389/fpubh.2022.984469

**Published:** 2022-10-06

**Authors:** Mohamed Ahmed Said, Majed M. Alhumaid, Ibrahim I. Atta, Khairi Mahmoud Al-Sababha, Mohamed Abdelmoneim Abdelrahman, Mohammed Shaab Alibrahim

**Affiliations:** ^1^Department of Physical Education, College of Education, King Faisal University, Al-Ahsa, Saudi Arabia; ^2^Higher Institute of Sport and Physical Education of Kef, Jendouba University, Jendouba, Tunisia

**Keywords:** children, adolescents, body mass index (BMI), health-related physical fitness (HRPF), fat-to-lean ratio

## Abstract

**Background:**

Several studies suggest that health-related physical fitness may play a prominent role in preventing obesity in children and adolescents.

**Objectives:**

The present study examined fitness levels using five components of health-related fitness in Saudi students aged 10–17 years (fat-to-lean mass ratio, cardiorespiratory endurance, upper body strength and endurance, abdominal muscle strength and endurance, and flexibility). Subsequently, the association between BMI and a health-related fitness index (HR-PFI) based on the five fitness components was investigated.

**Methods:**

The study was conducted on 1,291 students with a mean age of 12.95 ± 1.72 years. Participants included 1,030 boys aged 12.80 ± 1.79 years, with 479 young boys (11.24 ± 0.81b years), and 551 adolescents (14.16 ± 1.21 years). Moreover, the study examined 261 girls averaging 13.54 ± 1.2 years old, with 66 young girls (11.92 ± 0.27 years), and 195 teenage girls (14.09 ± 0.85 years). Each participant's health-related fitness level was assessed by the following tests: Bioelectrical Impedance Analyzer (BIA) for body composition, one-mile run/walk test for cardiorespiratory endurance, curl-up test for abdominal muscle strength and endurance (AMSE), push-up test for upper body strength and endurance (UBSE), and back-saver sit-and-reach test for flexibility.

**Results:**

The overall prevalence of overweight and obesity was 10.4 and 24.7% in boys and 10 and 8.4% in girls, respectively. The mean *Z-*scores of performances decreased from the underweight to the obese groups. BMI was positively associated with the ratio of fat mass to lean mass and negatively associated with cardiorespiratory endurance in the overall group of participants as well as in the subgroups by sex and age categories. BMI was also negatively associated with flexibility and HR-PFI in the total group, UBSE, AMSE, and HR-PFI in prepubertal boys, and UBSE in prepubertal girls. The coefficient of determination values was 0.65 in the total group, 0.72 in prepubertal boys, 0.863 in adolescent boys, 0.956 in prepubertal girls, and 0.818 in adolescent girls.

**Conclusions:**

Overall health-related physical fitness, fat-to-lean mass ratio, and cardiorespiratory endurance are the factors that most affect BMI in Saudi students aged 10 to 17.

## Introduction

Evidence shows that health-promoting behaviors, such as adequate sleep, healthy eating, regular physical activity, and maintaining healthy body weight and mental and social status, promote health and well-being early in life and are associated with higher levels of healthy behaviors in adulthood. These behaviors prevent or delay the onset of many life-threatening diseases and chronic conditions later in life, including cardiovascular disease, developmental disabilities, diabetes mellitus, and obesity (1). Skills are acquired and lifestyle habits are built when young, the effects of which will be perceived in early adulthood. Thus, establishing a healthy lifestyle is essential based on developing environments conducive to health, strengthening protective factors, and acquiring skills promoting the adoption of behaviors that promote health.

Relevant studies indicated that obese adults are at higher risk of developing morbid obesity, premature death, and disability if they were obese as children (2). In addition, childhood obesity could be associated with several health problems such as difficulty breathing, osteoporosis, hypertension, early markers of cardiovascular disease, diabetes type II, and psychosocial disorders (3). A meta-analysis published by *THE LANCET*, evaluating individual-participant data for 10,6 million adults in 239 prospective cohort studies in 32 countries located on four continents (Asia, Australia, Europe, and North America), noted that all-cause mortality was minimal at BMI between 20.0 and 25.0 kg/m^2^, however, it increased approximately log-linearly with BMI over 25.0 kg/m^2^. The authors concluded that wherever overweight and obesity are common, their associations with higher all-cause mortality are broadly similar in different populations, supporting strategies to address the full spectrum of excessive adiposity globally (4).

Raghuveer et al. (5) identified that physical activity (PA) is one of the most effective tools for treating and preventing health risk factors. The authors' findings indicated strong associations between PA and physical inactivity in childhood and adolescence and their short- and long-term health effects. It has also been recognized that PA is related to physical fitness (PF), and therefore physically active children will have higher levels of fitness. Studies have confirmed the existence of a significant relationship between PA, level of PF, and the risk of overweight and obesity in school-aged children and youth (6). It has also been reported that children and adolescents less engaged in regular PA and with a low level of fitness are more likely to gain weight over time than those with a high level of fitness (7–9).

The *American College of Sports Medicine Guidelines* suggested in its tenth edition that people should focus on a variety of mechanisms that improve overall fitness to improve adherence to physical activity guidelines developed by health organizations. Although physical fitness encompasses a wide range of physical attributes (components), five health-related components are primarily discussed in the physical activity literature. These health additives reflect an individual's health status, encompassing cardiorespiratory endurance, muscular strength, flexibility, and body composition (10).

Cardiorespiratory endurance, typically quantified as maximal oxygen uptake (VO_2_ max), is the component of PF that most assures ongoing PA involvement and predicts various health indicators, such as health cardiometabolic, and premature cardiovascular disease, school and mental performance (11). Muscular strength and endurance are associated with improved quality of life and the ability to perform functional tasks without exhaustion. Maintaining muscle, bone, and joint strength allows for better movement and reduces the risk of osteoporosis and falls in the elderly (12). Flexibility is also a factor that affects the ability of individuals to vigorously carry out their daily activities. Maintaining adequate levels of flexibility improves the supply of blood and nutrients to cartilage and other joint structures and increases the amount of synovial fluid, which delays muscle fatigue, prevents postural problems, and reduces the incidence of muscle injuries, especially in the lower back (13). Body composition is the final component of health-related fitness. A healthy body composition contains less body fat and more lean mass (LM), which includes muscle, bone, and organs. Recent studies have shown that different body compositions, including fat mass and lean mass, may play distinct roles in health outcomes (14).

BMI is commonly used to assess obesity. However, one of the major drawbacks of BMI is that it does not reflect body composition. BMI is unable to differentiate between fat mass and lean mass, so individuals with the same BMI can have very different body compositions (15, 16). In children and adolescents, the BMI changes significantly during growth and development and does not differentiate between fat mass and lean mass. The use of BMI can lead to important classification errors, particularly when the pathology associated with obesity depends on fat mass. Therefore, the ideal monitoring tool should record obesity directly (17). The fat-to-lean mass ratio has been proposed as a potential new indicator to assess the combined effect of body compositions (15). Prado et al. (18) proposed that the fat-to-lean mass ratio represents a metabolic load/capacity model. The model employs fat mass as the metabolic load factor defined as the extent of damage to a system, while lean mass represents the metabolic load factor using a counteroffensive system capacity (18). A higher fat-to-lean mass ratio has also been proposed as an alternative definition of sarcopenic obesity, which has been characterized as a confluence of sarcopenia and obesity (19).

In addition to the critical limitations of using BMI as an indicator of body composition and the need to use other alternatives such as the fat-to-lean mass ratio, health-related fitness studies have used relatively few fitness tests as a proxy for fitness, which have not allowed for a comprehensive assessment of fitness levels (20) or have focused on performance-related components and therefore the results obtained do not reflect the health status of the study population. Moreover, although there are many studies associating BMI with physical activity, few have investigated the relationship between BMI status and physical fitness, particularly among school-age subjects (20). The present study aims to (1) determine fitness levels using the five health-related physical fitness components (fat-to-lean mass ratio, cardiorespiratory endurance, upper body strength and endurance, abdominal muscle strength and endurance, and flexibility) in Saudi students aged 10–17 years; and (2) investigate the relationship between BMI and a health-related fitness index (HR-PFI) based on the five components of fitness.

## Methods

### Participants

A sample of 5,000 Saudi children and adolescents (2,500 boys and 2,500 girls) was selected using a random geographic cluster sampling technique from middle and high schools of the governorate of Al-Ahsa, Saudi Arabia (21). Positive responses received from school officials and physical education teachers included 3,029 students (2,450 males and 579 females), while the remainder declined to participate. Disapproval was mostly attributed to lack of physical education in the school curriculum, especially in girls' schools, lack of adequate infrastructure, including well-equipped gymnasiums, and cultural reasons that discourage participation in physical activity. Before the measurements, students who met one or more criteria from the following list were excluded: Age <10 or >17 years, BMI value <10 or >40 kg/m^2^, or any contraindication to physical activity. Failure to complete one or more of the study tests, and the lack of informed and signed consent by participants over 16 or parents under 16 were also considered exclusion criteria. After measurements, results were subsequently checked for outliers with the 1.5 ^*^ interquartile range rule, and all owners were excluded. Extreme and outlier values were <21 and >35 cm for the back-saver sit-and-reach test, <320 and >938 s for the one-mile run/walk test, 0 and > 30 units for the push-up test, and 0 and > 40 units for the curl-up test. The final sample retained for analysis was 1,291 students (1,030 boys and 261 girls) aged 10–17. Participants were categorized according to the WHO classification into two sex-specific- age groups (22): (1) children (<13 years), and (2) adolescents (>13 years). They were also categorized into four BMI groups according to the age and sex-specific cut-off values suggested by the WHO 2007 growth reference (5–19 years): (1) underweight (UW; BMI-for-age <5th percentile), normal weight (NW; 5th percentile ≤ BMI-for-age <85th percentile), overweight (OW; 85th percentile ≤ BMI-for-age <95th percentile) and obese [OB; BMI-for-age ≥95th percentile; (23)]. This study was approved by the Ethical Committee of the Deanship of Scientific Research, King Faisal University (Ref. No. KFU-REC-2021-OCT-EA00019).

### Procedures

Health-related fitness level was assessed by the following tests: Bioelectrical Impedance Analyzer (BIA) for body composition, One-mile run/walk test for cardiorespiratory endurance, the curl-up test for abdominal muscle strength and endurance (AMSE), the push-up test for upper body strength and endurance (UBSE), and the back-saver sit-and-reach test for flexibility. Assessments took place in the school gymnasium, at least 2 postprandial hours during two class periods of the school day (10–12 a.m.), and on two separate days to eliminate any possible fatigue effects. All students were organized into pairs, one tests the other without any performance assistance, familiarized with the test protocols in the last physical education session, and briefed on the criteria for success and how to record the scores on the sheet. All assessments were conducted by physical education teachers under the supervision of a member of our research team. Students who regularly participated in sports activities were asked to refrain from this training the day before and during the assessment sessions. All participants were instructed to avoid sleep deprivation, eat a light meal at least 3 h prior to the test, and ensure that they were adequately hydrated before and during the testing sessions (11). In addition to the testing section, each session included a detailed explanation of the testing procedure, a 10-min warm-up period, and a 5-min cool-down. Body composition and the one-mile run test were administered on day 1 and the remaining three tests were given on day 2. A 3–5-min recovery period was allowed between tests, which were performed in the same order in all schools.

### Study outcomes

#### Anthropometry

Each participant's height was measured to the nearest 0.1 cm using a stadiometer (Holtain, Crymych, Wales, UK). Body composition estimates were obtained by bioelectric impedance analysis technology using a Tanita Segmental Body Composition Monitor (FitScan BC-601, Japan). All measurements were performed according to the manufacturer's guidelines, with minimal clothing, clean feet, and under consistent hydration conditions. For children aged 5–17, only weight, percentage of body fat (PBF), and body mass index (BMI) were displayed. BMI was calculated as weight (kg) / height (m^2^), fat mass was calculated as weight (kg) × body fat percentage, and fat-free mass was determined as weight (kg) – fat mass (kg). The fat-to-lean mass ratio was used as an indicator of body composition.

#### Cardiorespiratory endurance: The one-mile walk/run test

The one-mile walk/run test was administered in a gymnasium on a 200-m long track prepared in advance for the event. Participants were instructed to run 1 mile as fast as they can. Walking was allowed; however, stopping, sitting, or leaving the track was prohibited. The 1-mile run/walk time (MRWT) represents the time from the start to the end of the 1-mile distance, recorded by the physical education teacher using a digital quartz stopwatch (FINIS 3X300M; California, USA). Aerobic capacity (VO_2_max) was estimated using Cureton et al.'s (24) formula: VO_2_max = −8.41 (MRWT) + 0.34 (MRWT)^2^ + 0.21 (Age × Gender) −0.84 (BMI) + 108.94, with VO_2_peak (ml.kg^−1^.min^−1^) from gender (0 = F, 1 = M), age (yrs.), body mass index (kg.m^−2^; BMI) and MRWT (min).

#### Abdominal muscular strength and endurance: The curl-up test

For this test, the participant lies in a supine position on the mat with knees bent at an angle of ~140°, feet flat on the floor, arms outstretched and parallel to the trunk, and hands flat on the floor. A strip of cardboard measuring 80 × 11.43 cm is placed under the legs so the fingertips touch the edge closest to the measuring tape. Keeping the heels flat on the floor, the participant slowly curls up, sliding their fingers along the measuring tape until it touches the far edge, then curls up to the position of departure.

Before starting the test, the subject's partner should ensure that the participant's shoulders are in the normal resting position, fingers stretched out, and the head is in contact with the mat. The participant was encouraged to do as many curl-ups as possible. Execution must be continuous, with breaks and rest periods forbidden. The test ends when the participant completes 75 curl-ups, a second form correction is made, or the subject cannot continue. The count is made by the participant's partner when the subject's head returns to the mat. The score is the number of successful curl-ups.

#### Upper body strength and endurance (UBSE): The push-up test

From the supine position with the hands placed flat under or slightly wider than the shoulders, fingers stretched out, legs straight and slightly apart from each other and toes bent below, the participant pushes upward with the arms until they reach their full extension, keeping the legs and back straight. The participant then lowers their body using their arms in a position where the arms are parallel to the floor and the elbows bent at a 90-degree angle. The participant should perform as many 90° push-ups as possible in a continuous rhythm of motion, with the back kept in a straight line from head to toe, and without breaks or rest periods. The test ends when the participant makes two technical errors or appears to be in extreme discomfort or pain, and the score will be the number of 90° push-ups performed correctly.

#### Flexibility: Back-saver sit-and-reach test

The participant sits barefoot on the floor. One leg is extended and placed flush with a measuring box and the other leg is flexed with the foot flat on the floor. A measuring scale was placed on top of the box so that the vertical plane against which the subject's foot was placed was exactly at the 23-cm mark. The “zero” end of the measurement ruler is closest to the participant, who bends forward and slides their hands placed on top of each other along the measurement line as far as possible. Ballistic or rebounding movements are prohibited, and the maximum score allowed is 12 inches (30.48 cm). After three training attempts, the fourth reach is held for at least one second to record the distance. The score is recorded to the nearest centimeter as the distance reached by the fingertips.

### Data analyses

Statistical analysis was performed with SPSS V.26. Data were presented as mean ± standard deviation for quantitative variables and frequencies and percentages for qualitative variables. Differences between groups were tested using a chi-square test, *t*-test, or one-way analysis of variance (ANOVA), with Bonferroni *post hoc* testing for subsequent pairwise comparisons for k-independent samples. *Z-*scores were calculated for each item using gender- and age-specific means and standard deviations. In addition, *Z-*scores were inverted for the fat-to-lean mass ratio, as lower values reflect better performance. Each participant's *Z-*scores were summed to obtain a health-related physical fitness index [HR-PFI; (25)]. Normality and homoscedasticity of the residuals were checked. The multicollinearity of the independent variables was verified using the variance inflation factor (VIF), indicating that the model exhibits strong fat-to-lean mass, cardiorespiratory endurance, and HR-PFI multicollinearity. Principal components analysis was used to address collinearity between variables. In addition, a multiple linear regression procedure was performed to determine the extent of variation in BMI that could be explained by the components derived from principal component analysis. Effects are reported as unstandardized coefficients (β and standard error), R-squared, t-statistic, and 95.0% confidence interval for β.

## Results

### Anthropometric characteristics

Overall, 1,291 students aged 12.95 ± 1.72 years completed all stages of the study. Subjects included 1,030 boys with an average age of 12.80 ± 1.79 years, including 479 young boys (11.24 ± 0.81b years) and 551 adolescents (14.16 ± 1.21 years). In addition, 261 girls participated in the study averaging 13.54 ± 1.2 years, with 66 young girls (11.92 ± 0.27 years) and 195 teenage girls (14.09 ± 0.85 years). The sex-specific BMI-for-age classification indicated a prevalence of 10.4% overweight and 24.7% obesity in boys and 10 and 8.4% in girls, respectively. Significant differences were noted between boys and girls (χ^2^ = 69.894; *p* = 0.001) and between female children and their adolescent peers (χ^2^ = 24.815; *p* = 0.001). Table 1 provides additional relevant information regarding the prevalence of overweight and obesity among participants by gender and age category.

**Table 1:** Prevalence of underweight, normal weight, overweight, and obesity among participants stratified by sex and age category.

		**Boys**			**Girls**		
		**Total** **(*n =* 1030)**	**Children** **(*n =* 479)**	**Adolescents** **(*n =* 551)**	**Total** **(*n =* 261)**	**Children** **(*n =* 66)**	**Adolescents** **(*n =* 195)**
Body Mass Index	Underweight	134 (13.0)	62 (12.9)	72 (13.0)	33 (12.6)	5 (7.6)	28 (14.4)
	Normal weight	535 (51.9)	237 (49.5)	298 (54.0)	180 (69.0)	43 (65.2)	137 (70.3)
	Overweight	107 (10.4)	52 (10.9)	55 (10.0)	26 (10.0)	14 (21.2)	12 (6.2)
	Obese	255 (24.7)	128 (26.7)	127 (23.0)	22 (8.4)	4 (6.1)	18 (9.2)
Between sex-age groups		χ^2^ *=* 69.894; *p =* 0.001					
Between age groups			χ^2^ *=* 2.555; *p =* 0.465			χ^2^ *=* 24.815; *p =* 0.001	

Data also revealed that girls were generally taller, heavier, and fatter than boys but with similar build and lean mass. Male children had the lowest values for all anthropometric variables except BMI (*p* < 0.01 for male adolescents for fat-to-lean mass ratio; *p* < 0.001 for others), whereas the highest values were observed in adolescent boys for lean mass and in their female counterparts for height, PBF, fat mass, and fat-to-lean mass ratio. A significant difference was also noted in BMI (*p* < 0.001) and fat-to-lean mass (*p* < 0.05) between the two groups of boys and in height between the two groups of girls (*p* < 0.05). No significant difference was noted between the two groups of girls by age category in body composition (Table 2).

**Table 2:** Anthropometric characteristics of participants stratified by sex and age category.

	**Participants (*n =* 1,291)**	**Boys**			**Girls**			**Between sex-age groups**	
		**Total boys** **(*n =* 1030)**	**Children** **(*n =* 479)**	**Adolescents** **(*n =* 551)**	**Total girls** **(*n =* 261)**	**Children** **(*n =* 66)**	**Adolescents (*n =* 195)**	***f***	***p***
Height (cm)	149.17 ± 12.66	147.56 ± 13.13	139.04 ± 8.65^b,c,d^	154.97 ± 11.86^a^	155.40 ± 7.77^***^	152.30 ± 6.37^a,d^	156.45 ± 7.95^a,c^	266.09	0.001
Weight (kg)	43.8 ± 13.67	43.07 ± 14.31	36.85 ± 11.74^b,c,d^	48.07 ± 13.58^a,c^	46.57 ± 10.24^***^	43.4545 ± 6.93^a,b^	47.62 ± 10.95^a^	80.61	0.001
BMI (kg/m^2^)	19.39 ± 4.49	19.43 ± 4.69	18.80 ± 4.68^b^	19.98 ± 4.64^a^	19.23 ± 3.60	18.72 ± 2.65	19.41 ± 3.86	6.509	0.001
PBF (%)	22.02 ± 5.97	21.07 ± 5.99	20.41 ± 6.08^b,c,d^	21.64 ± 5.8^a,c,d^	25.78 ± 4.12^***^	25.31 ± 3.38^a,b^	25.93 ± 4.33^a,b^	52.83	0.001
Fat Mass (kg)	10.3 ± 5.94	9.78 ± 6.09	8.16 ± 5.20^b,c,d^	11.19 ± 6.46^a,d^	12.33 ± 4.78^***^	11.18 ± 3.09^a^	12.71 ± 5.18^a,b^	39.39	0.001
LM (kg)	33.49 ± 8.28	33.29 ± 8.78	28.70 ± 6.84^b,c,d^	37.29 ± 8.32^a,c,d^	34.25 ± 5.85	32.27 ± 4.04^a,b^	34.91 ± 6.21^a,b^	120.93	0.001
Fat-to-lean mass ratio	0.29 ± 0.106	0.275 ± 0.106	0.264 ± 0.105^b,c,d^	0.28 ± 0.107^a,c,d^	0.352 ± 0.08^***^	0.342 ± 0.062^a,b^	0.356 ± 0.085^a,b^	43.57	0.001

Data are means ± standard deviation.

*** p < 0.001 differs from boys using T-test;

a differs from male children;

b differs from male adolescents;

c differs from female children;

d differs from female adolescents using ANOVA. BMI, Body mass index; PBF, Percentage body fat; LM, Lean mass.

### Health-related fitness

Table 3 shows the performance achieved in all physical tests and the correspondent HR-PFI of participants by sex and age category. A student's *t*-test showed significant superiority of males over females in all tests except flexibility, where females performed significantly better than males (*p* < 0.001 for all). However, the HR-PFI indicated that the fitness level would not differ at all between boys and girls. Conversely, when reported to gender and age categories, the *post hoc* test revealed a significant difference between the groups of boys compared to those of girls in cardiorespiratory endurance, UBSE, and AMSE (*p* < 0.01 for groups of boys vs. prepubescent girls in push-up test and for adolescent boys vs. prepubescent girls in Curl-up test; *p* < 0.001 for the rest). The adolescent boys seemed also less flexible than the other three groups (*p* < 0.01 for prepubescent girls; *p* < 0.001 for the rest), and have better cardiorespiratory endurance than their children peers (*p* < 0.01; Table 3).

**Table 3:** Performances in physical fitness tests of participants stratified by sex and age category.

	**Participants (*n =* 1291)**	**Boys**			**Girls**			**Between sex-age groups**	
		**Total boys** **(*n =* 1030)**	**Children** **(*n =* 479)**	**Adolescents** **(*n =* 551)**	**Total girls** **(*n =* 261)**	**Children** **(*n =* 66)**	**Adolescents** **(*n =* 195)**	**f**	**p**
One Mile (second)	636.05 ± 115.3	621.34 ± 116.92	622.00 ± 80.28^c,d^	620.76 ± 141.33^c,d^	694.08 ± 87.26^***^	700.92 ± 59.6^a,b^	691.76 ± 94.83^a,b^	29.586	0.001
VO_2_max (ml/kg/min)	46.25 ± 4.54	45.97 ± 4.78	45.47 ± 4.5^b,c,d^	46.40 ± 4.98^a,c,d^	41.72 ± 3.17 ^***^	41.70 ± 2.38^a,b^	41.73 ± 3.40^a,b^	65.915	0.001
Push-Up (unit)	11.81 ± 6.18	12.48 ± 6.27	12.39 ± 5.04^c,d^	12.56 ± 7.18^c,d^	9.17 ± 5.03 ^***^	9.80 ± 3.69^a,b^	8.96 ± 5.40^a,b^	21.294	0.001
Curl-Up (Unit)	19.09 ± 9.93	20.04 ± 10.25	20.35 ± 9.24^c,d^	21.14 ± 13.88^c,d^	15.38 ± 7.51 ^***^	15.29 ± 5.72^a,b^	15.41 ± 8.04^a,b^	16.144	0.001
Flexibility (cm)	27.99 ± 3.46	27.70 ± 3.60	28.62 ± 3.01^b^	26.91 ± 3.88^a,c,d^	29.10 ± 2.54 ^***^	28.49 ± 3.24^b^	29.3 ± 2.22^b^	35.620	0.001
HR-PFI	−0.055 ± 3.403	−0.0756 ± 3.457	−0.004 ± 3.801	−0.138 ± 3.13	0.0236 ± 3.19	0.007 ± 3.33	0.029 ± 3.148	0.19	0.903

Data are means ± standard deviation.

*** p < 0.001 differs from boys;

a differs from male children;

b differs from male adolescents;

c differs from female children;

d differs from female adolescents using ANOVA. VO_2_max, Maximal oxygen consumption; HR-PFI, Health-related physical fitness index.

Table 4 shows the mean *Z-*scores for fat-to-lean mass ratio, cardiorespiratory endurance, UBSE, AMSE, and flexibility as well as the HR-PFI level of boys stratified by BMI category. All fitness components showed significant between-group differences (*F* = 1,117.251, 550.813, 82.960, 53.330, and 4.808, respectively; *p* < 0.01 for flexibility and *p* < 0.001 for the remainder), and *post hoc* testing showed that values decreased in the order of UW, NW, OW, and OB groups for all fitness components (*p* < 0. 001 for all), with no significant differences between the UW and NW groups for AMSE and the UW and NW groups vs. the OW group and the OW group vs. the OB group for flexibility. The HR-PFI also showed a significant intergroup difference (*F* = 417.6, *p* < 0.001), and the *post hoc* test asserted that the lowest value was recorded in the OB group (*p* < 0.001 for all) and the highest in the UW group, with significant differences between all BMI groups (*p* < 0.001 for all).

**Table 4:** Mean *Z-*score of health-related physical fitness components of male participants by BMI category.

	**Boys (*N =* 1030)**				**Between** **BMI-groups**	
	**UW (*N =* 134)**	**NW (*N =* 534)**	**OW (*N =* 107)**	**OB (*N =* 255)**	***F***	***P*-value**
Fat-to-lean mass ratio	0.974 ± 0.179^b,c,d^	0.454 ± 0.284^a,c,d^	−0.185 ± 0.341^a,b,d^	−1.474 ± 0.821^a,b,c^	1,117.251	0.001
Cardiorespiratory endurance	1.046 ± 0.547^b,c,d^	0.39 ± 0.566^a,c,d^	−0.278 ± 0.46^a,c,d^	−1.25 ± 0.796^a,b,c^	550.813	0.001
Upper body strength and endurance	0.53 ± 1.03^b,c,d^	0.25 ± 0.94^a,c,d^	−0.255 ± 0.86^a,c,d^	−0.693 ± 0.736^a,b,c^	82.96	0.001
Abdominal strength and endurance	0.318 ± 1.01^c,d^	0.130 ± 0.877^c,d^	−0.146 ± 0.877^a,c,d^	−0.593 ± 0.6^a,b,c^	53.33	0.001
Flexibility	0.093 ± 0.988^d^	0.07 ± 0.923^d^	−0.102 ± 0.949	−0.203 ± 1.148^a,b^	4.808	0.002
HR_PFI	2.878 ± 2.450^b,c,d^	1.094 ± 2.283^a,c,d^	−1.350 ± 2.509^a,c,d^	−4.239 ± 2.719^a,b,c^	417.6	0.001

Results are expressed as mean ±SD. UW, Underweight; NW, Normal weight; OW, Overweight; OB, Obese; HR-PFI, Health-related physical fitness index.

a Differs significantly from UW;

b differs significantly from NW;

c differs significantly from OW;

d differs significantly from OB.

**Figure 1:**
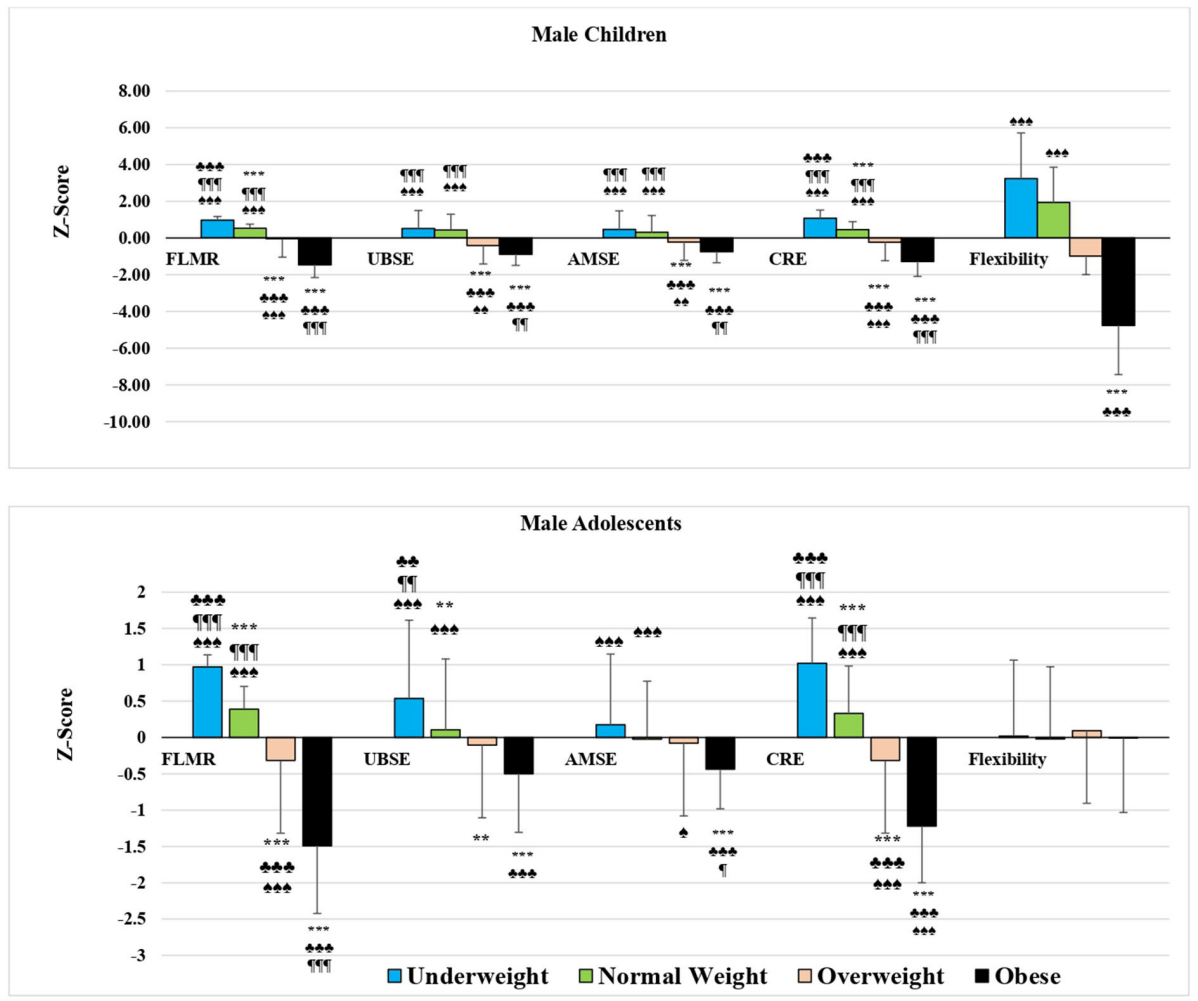
Mean *Z*-score of health-related physical fitness components in male students by age and BMI category. FLMR, Fat-to-lean mass ratio; UBSE, Upper body strength and endurance; AMSE, Abdominal muscular strength and endurance; CRE, Cardio-respiratory endurance. ^*^p < 0.05, **p < 0.01, ***p < 0.001 differs from UW; ♣*p* < 0.05, ♣♣*p* < 0.01, ♣♣♣*p* < 0.001 differs from NW; ¶*p* < 0.05, ¶¶*p* < 0.01, ¶¶¶*p* < 0.001 differs from OW; ♠*p* < 0.05, ♠♠*p* < 0.01, ♠♠♠*p* < 0.001 differs from OB using ANOVA.

All fitness component values also decreased when moving from the underweight to the obese group in the age and BMI category groups of boys, except for flexibility in adolescents in whom no significant differences were noted between all BMI groups. In addition, the significant differences previously recorded in prepubescent boys between the UW and NW groups in UBSE and AMSE, and in adolescent groups between the OW group vs. the NW and OB groups in UBSE and between the OW group vs. the UW and NW groups in AMSE were not retrieved (Figures 1, **3A**).

**Figure 2:**
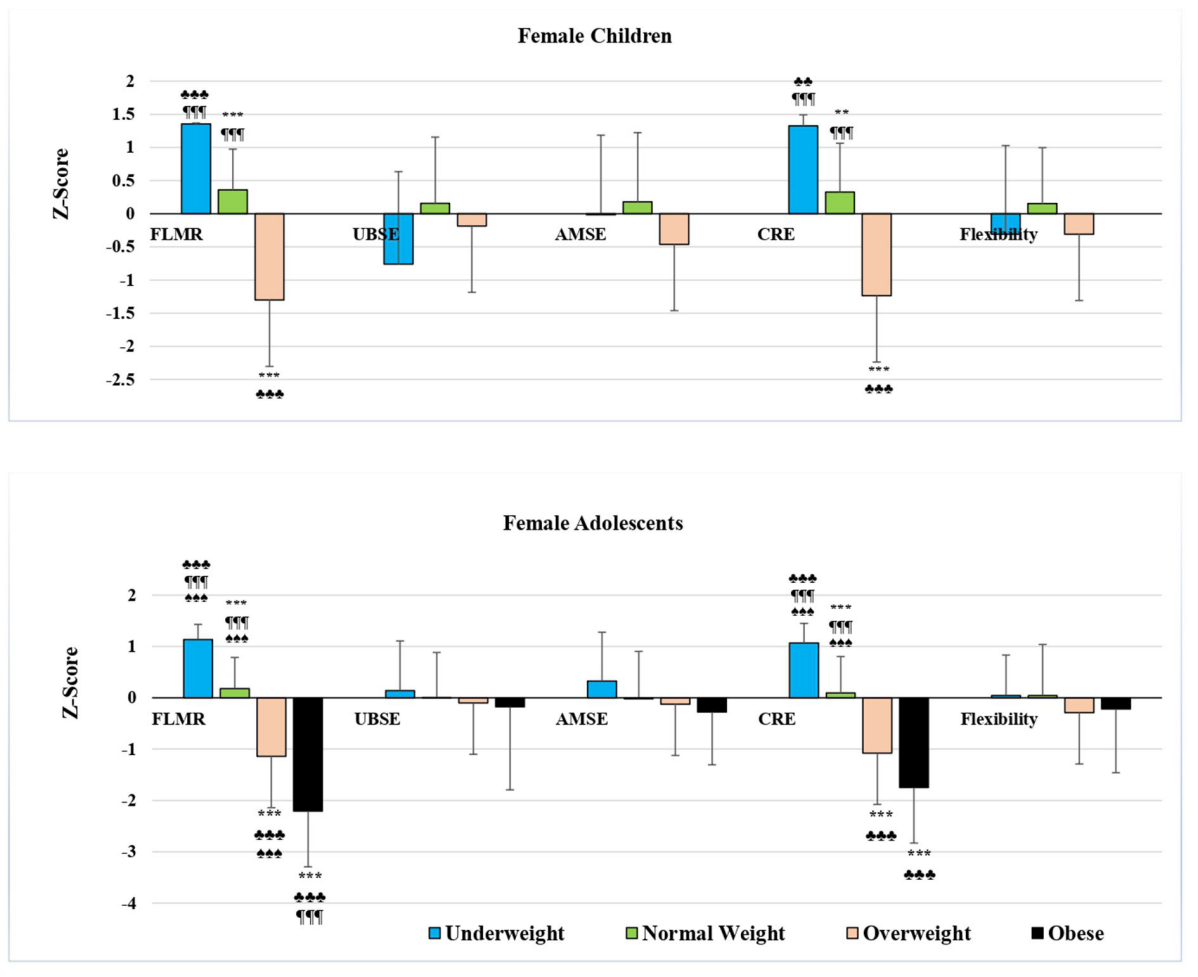
Mean Z-score of health-related physical fitness components in female students by age and BMI category. FLMR, Fat-to-lean mass ratio; UBSE, Upper body strength and endurance; AMSE, Abdominal muscular strength and endurance; CRE, Cardio-respiratory endurance *p < 0.05, **p < 0.01, ***p < 0.001 differs from UW; ♣*p* < 0.05, ♣♣*p* < 0.01, ♣♣♣*p* < 0.001 differs from NW; ¶*p* < 0.05, ¶¶*p* < 0.01, ¶¶¶*p* < 0.001 differs from OW; ♠*p* < 0.05, ♠♠*p* < 0.01, ♠♠♠*p* < 0.001 differs from OB using ANOVA.

**Figure 3:**
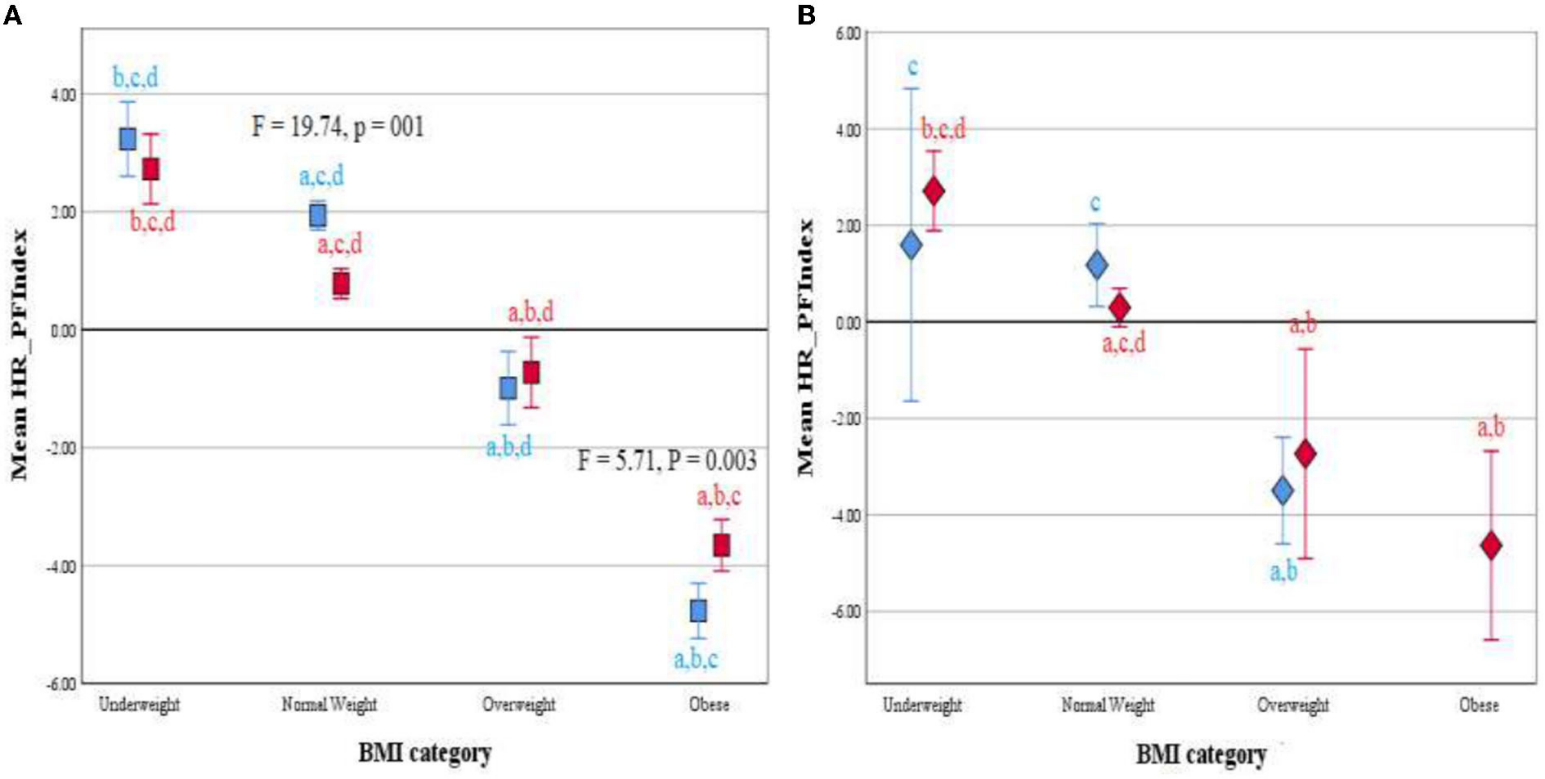
Clustered Error Bar Mean of HR-PFI by BMI category and age group (Children, blue; adolescents, red) in male **(A)** and female **(B)** participants. Health-related physical fitness index, HR-PHI. ^a^differs from Underweight; ^b^differs from Normal weight; ^c^differs from Overweight; ^d^differs from Obese using ANOVA.

In girls, the mean standard scores for the fat-to-lean mass ratio, cardiorespiratory endurance, and HR-PFI showed significant intergroup differences (*F* = 161.843, 94.572, and 45.525, respectively; *p* < 0. 001 for all), and *post hoc* tests showed significant differences between all groups of girls by BMI category except for the OW and OB groups in HR-PFI (Table 5; *p* < 0. 05 for OW vs. OB in cardiorespiratory endurance and *p* < 0.001 for the rest), and between the groups of girls by age and BMI category, except for the prepubescent UW and NW groups in HR-PFI and for the adolescent OW and OB groups in cardiorespiratory endurance and HR-PFI (Figures 2, 3B; *p* < 0.001 for all). Values decreased from the underweight to the obese groups of girls, and no significant differences were noted in the other fitness components.

**Table 5:** Mean *Z-*score of health-related physical fitness components of female participants by BMI category.

	**Girls (*N* = 261)**				**Between BMI-groups**	
	**UW (*N =* 33)**	**NW (*N =* 182)**	**OW (*N =* 29)**	**OB (*N =* 17)**	***F***	***P*-value**
Fat-to-lean mass ratio	1.17 ± 0.28^b,c,d^	0.224 ± 0.61^a,c,d^	−1.235 ± 0.504^a,c,d^	−2.208 ± 1.085^a,b,c^	161.843	0.001
Cardiorespiratory endurance	1.106 ± 0.37^b,c,d^	0.149 ± 0.723^a,c,d^	−1.171 ± 0.448^a,c,d^	−1.749 ± 1.082^a,b,c^	94.572	0.001
Upper body strength and endurance	0.0035 ± 1.075	0.04 ± 0.92	−0.15 ± 0.973	−0.178 ± 1.632	0.492	0.688
Abdominal strength and endurance	0.274 ± 0.98	0.028 ± 0.956	−0.322 ± 1.18	−0.278 ± 1.03	2.363	0.072
Flexibility	−0.009 ± 0.875	0.07 ± 0.961	−0.301 ± 1.184	−0.22 ± 1.24	1.456	0.227
HR_PFI	2.545 ± 2.199^b,c,d^	0.509 ± 2.506^a,c,d^	−3.18 ± 2.715^a,b^	−4.632 ± 3.806^a,b^	45.525	0.001

Results are expressed as mean ±SD. UW, Underweight; NW, Normal weight; OW, Overweight; OB, Obese; HR-PFI, Fat mass, Health-related physical fitness index. adiffers significantly from UW; bdiffers significantly from NW; cdiffers significantly from OW; ddiffers significantly from OB.

### Partial component analysis

Using the scree plot and considering only eigenvalues >1.0 as significant, after Varimax rotation with Kaiser Normalization, the principal components and the generated varimax factors (VFs) are shown in Table 6. In the present study, only strong VFs (> 0.75) were considered for selection and interpretation due to their significant factor loadings (26). In the total sample, BMI was negatively associated with the first principal component (β = −3.6175) and positively associated with the second component (β = 0.205). The proportion of the variance in BMI that can be explained by these components is approximately 65%, and the selected VFs are fat-to-lean mass ratio (factor loading = −0.886), cardiorespiratory endurance (factor loading = 0.891), and HR-PFI (factor loading = 0.873) in C1 and flexibility (factor loading = 0.896) in C2.

**Table 6:** Factors extracted, eigenvalues and proportion of variance after rotation.

**Component**			**Boys**			**Girls**				
	**All participants (*N =* 1291)**		**Children (*N =* 479)**	**Adolescents** **(*N =* 551)**		**Children (*N =* 66)**		**Adolescents (*N =* 195)**		
	**1**	**2**	**1**	**1**	**2**	**1**	**2**	**1**	**2**	**3**
Fat-to-lean mass ratio	**−0.886**	00.108	**−0.867**	**−0.924**	−0.109	**−0.969**	−0.139	**−0.967**	−0.088	−0.012
Cardiorespiratory endurance	**0.891**	−0.169	**0.876**	**0.929**	0.084	**0.961**	0.133	**0.967**	0.094	0.016
Upper body strength and endurance	0.704	0.309	**0.841**	0.356	0.714	−0.024	**0.829**	0.086	**0.917**	−0.052
Abdominal strength and endurance	0.660	0.366	**0.785**	0.267	0.745	0.357	0.588	0.106	**0.912**	0.032
Flexibility	0.017	**0.896**	0.417	−0.183	0.663	0.148	0.620	0.045	0.002	**0.999**
HR_PFI	**0.873**	0.430	**0.996**	0.715	0.691	0.721	0.693	0.694	0.643	0.322
Eigenvalues after varimax rotation	3.272	1.257	4.01	2.459	2.001	2.533	1.934	2.373	2.103	1.105
% Cumulative	54.531	75.476	66.78	40.98	74.337	42.213	74.448	39.549	74.599	93.017

Extraction Method: Principal Component Analysis. Rotation Method: Varimax with Kaiser Normalization. Retained eigenvalue of the factors >1. Retained VF of the factor loading >0.75 (bold). HR-PFI, Health-related physical fitness index.

In prepubescent boys, only one component was extracted in which fat-to-lean mass ratio, cardiorespiratory endurance, UBSE, AMSE, and HR-PFI were retained as strong VFs. The factor loadings were −0.867, 0.876, 0.841, 0.785, and 0.996, respectively. This component had a negative effect on the BMI of prepubescent boys with a β of −3.972, an R-squared of 0.72, and a cumulative percentage of 66.78%. On the contrary, two partial component factors were created in adolescent boys and both were negatively associated with BMI with a coefficient of determination of 0.863 (Table 7). In C1 (β = −4.291; *p* < 0.001), the fat-to-lean mass ratio (factor loadings = −0.924) and cardiorespiratory endurance (factor loading = 0.929) were retained, however, no strong VFs were observed in C2 (β = −0.406; *p* < 0.01).

**Table 7:** Multiple linear regression analysis for associations between BMI and physical fitness of Saudi students aged 10–17.

**Model**			**β (Std. Error)**	***R*-squared**	***t*-statistic**	**95.0% confidence** **Interval for B**	
						**Lower bound**	**Upper bound**
All participants (*N =* 1291)		Constant	19.394 (0.074)^***^	0.650	261.755	19.248	19.539
		REGR factor score 1	−3.617 (0.074)^***^		−48.796	−3.762	−3.471
		REGR factor score 2	0.205 (0.061)^**^		2.765	0.060	0.350
BOYS	Children (*N =* 479)	Constant	18.801 (0.113)^***^	0.720	165.971	18.579	19.024
		REGR factor score 1	−3.972 (0.113)^***^		−35.027	−4.195	−3.749
	Adolescents (*N =* 546)	Constant	19.983 (0.73)^***^	0.863	272.122	19.838	20.127
		REGR factor score 1	−4.291 (0.73)^***^		−58.375	−4.435	−4.146
		REGR factor score 2	−0.406 (0.73)^**^		−5.527	−0.551	−0.262
GIRLS	Children (*N =* 66)	Constant	18.719 (0.69)^***^	0.956	269.378	18.580	18.858
		REGR factor score 1	−2.558 (0.7)^***^		−36.532	−2.698	−2.418
		REGR factor score 2	−0.377 (0.7)^***^		−5.383	−0.517	−0.237
	Adolescents (*N =* 195)	Constant	19.413 (0.119)^**^	0.818	163.012	19.178	19.648
		REGR factor score 1	−3.489 (0.119)^***^		−29.224	−3.725	−3.254
		REGR factor score 2	0.65 (0.119)		0.544	−0.171	0.300
		REGR factor score 3	−0.148 (0.119)		−1.243	−0.384	0.087

Regression β coefficients (unstandardized) represent the degree of change in the BMI for every 1-unit of change in the predictor factors.

** *p* < 0.01,

*** *p* < 0.001. Std. Error = standard error.

In girls, PCA extracted two partial components with a cumulative 74.448% in prepubescents vs. three components with a cumulative 93.017% in adolescents (Table 6). Multiple linear regression analysis showed that in prepubescent girls BMI was negatively associated with both partial components (β = −2.558 and −0.377, respectively; *R*^2^ = 0.956), whereas it was only associated with the first factor in adolescents (β = −3.489; *R*^2^ = 0.818; Table 7). The retained VFs were fat-to-lean mass ratio and cardiorespiratory endurance at C1 (factor loadings = −0.969 and 0.961, respectively) and UBSE at C2 in prepubescent girls (factor loadings = 0.829), and fat-to-lean mass ratio and cardiorespiratory endurance at C1 (factor loadings = −0.965 and 0.967, respectively) in adolescent girls.

## Discussion

The current study aimed to examine the fitness level of Saudi students aged 10–17 years by gender and age groups, and then investigate the association that might exist between BMI and various health-related fitness components, separately or aggregated as an indicator (HR-PFI).

The main results indicated a prevalence of overweight and obesity of 10.4% and 24.7% in boys and 10% and 8.4% in girls, respectively. These findings are consistent with previous studies that have examined gender differences in childhood overweight and obesity and reported higher rates of overweight and obesity in boys than in girls (27, 28). Prepubescent boys had the lowest values for all anthropometric variables except BMI compared with the girls' groups, whereas the highest values were observed in adolescent boys for lean mass and in adolescent girls for height, PBF, fat mass, and fat-to-lean mass ratio. Significant inferiority was also noted in prepubescent boys compared to their adolescent peers in fat-to-lean mass ratio and in girls compared to their adolescent peers in height. There were no significant differences between the two groups of girls on any other variables.

Aljaadi and Alharbi (27) reported that overweight and obesity rates among Saudi children (5–19 years old) in 2010 were 31.6 and 14.3%, respectively. These rates increased in 2016 to 35.6 and 17.4%, respectively. Al Hussaini et al. (29) also reported overweight and obesity rates of about 12 and 18.4% among boys and 14.2 and 18% among girls among 7,930 6–16-year-old schoolchildren in the city of Riyadh, in the central region. They also noted that the prevalence of obesity was significantly higher in adolescents than in children (20.2 vs. 15.7%) and that overweight and obesity increased in conjunction with socioeconomic levels. However, Aliss et al. (30) found that approximately 40% of 5–15-year-old boys and 10% of girls were overweight or obese in the Jeddah region of western Saudi Arabia. Elkhodary and Farsi (31) also reported nearly 40% of overweight and obese boys and girls in the Jeddah region, with 18% of children and 16% of adolescents being overweight.

A higher prevalence of obesity in boys than in girls has also been previously reported in other countries (32). In a systematic review of 1769 surveys, reports, and studies published between 2008 and 2013, Ng et al. (33) reported a substantial increase in prevalence among children and adolescents in developed countries, with 23.8% of boys and 22.6% of girls who were overweight or obese in 2013. Canadian national data from 2004 to 2013 also showed a higher prevalence of obesity in older boys than in older girls (34). Similarly, in China, the prevalence of childhood obesity between 1985 and 2010 was consistently higher in boys aged 7-18 than in girls (35). In Poland, the prevalence of overweight and obesity increased between 2000 and 2010 among children aged 3 to 19, with a higher prevalence among boys than girls (36). According to Shah et al. (32), the higher prevalence of childhood obesity in boys compared to girls may be partly due to biological influences. Girls generally have a higher body fat percentage and lower lean body mass, which is associated with lower energy intake in girls than in boys. Studies have shown that sex steroid hormones are among the factors responsible for these differences in body composition in children and adolescents (37). Further, girls have higher circulating levels of leptin, a hormone that suppresses appetite and promotes energy consumption. Elevated serum leptin levels are a function of body fat and are directly proportional to levels of adiposity (38). Sociocultural factors may also influence differences in the prevalence of obesity between boys and girls. Recent studies suggest that girls, especially in high-income countries, prefer nutritious, low-calorie foods such as fruits and vegetables, while boys tend to eat more meat and calorie-dense foods (39, 40). Girls may also report higher levels of weight-related issues than boys, including a desire to lose weight, guilt over overeating, and low self-esteem. These socio-cultural differences are likely the result of gender stereotypes, as female identity is generally characterized by eating smaller portions and favoring healthier options for maintaining appearance, while food identity masculine is characterized by a feeling of satiety which emphasizes the optimization of physical performance (32).

Growth and maturation are factors that may explain the observed differences between prepubescent boys and the other sex-age groups, as well as the similarity between age groups of girls in anthropometric characteristics other than height (41). In the present study, all participants, regardless of gender, were 13 years old in chronological adolescence. However, the literature has clearly shown that individuals of the same age can differ markedly in terms of biological maturity. Biological maturation refers to the progression to a mature state and varies over time, rate, and between different body systems. There are significant differences between individuals in the level (extent of change), time (onset of change), and speed (rate of change) of biological maturation. Depending on these variables, children are considered to be biologically ahead of their chronological age (early individual), temporally ahead of their chronological age (middle individual), or behind their chronological age (late individual). The relative discrepancy and significant differences in biological maturation between children of the same chronological age underscore the limitations of using chronological age as a determinant in studies of school-age adolescents (42). Incorporating pubertal status would thus be useful to properly categorize the growth patterns in children with normal variants of puberty.

Data also revealed that, despite the lack of any significant difference in BMI between boys and girls, boys were more enduring, stronger, less fat, and less flexible than girls. According to Davis et al. (43), three physiological factors may explain these differences. First, although girls tend to store more total body fat and subcutaneous fat, they oxidize fat more than carbohydrates during endurance and high-intensity exercise due to differences in muscle fiber typology (i.e., the ratio of type I to type II fibers). Second, the smaller lungs and airways available to girls have been shown to influence factors important for physical performance, such as work of breathing and respiratory efficiency (44). Finally, compared to boys, girls may experience less metabolic stress at the muscle level during exercise, allowing them to better resist fatigue and require more rest periods. This allows girls to have less adaptive stimulus to physical activity, who tend to experience smaller relative gains and adjustments than boys in response to chronic exercise, despite equal workloads (45). Not surprisingly, girls, regardless of BMI, tend to have lower levels of fitness than their male counterparts (43).

Our data also noted that, when stratified by sex and age category, no significant differences between groups were noted in HR-PFI indicating relatively similar fitness levels in girls than in boys (−0.0756 vs. 0.0236). It is important to note that usually, boys are more fit than girls. This may partly explain the low prevalence of overweight and obesity observed in girls and confirmed by a significant association shown by multiple linear regression analysis between BMI and HR-PFI, the factor loadings of HR-PFI ranged from 0.694 to 0.996. However, when stratified by BMI category, the standard scores for boys indicated that raw score values were above average for the underweight and normal-weight groups and below average for the overweight and obese groups in the overall fitness index and all its components. Strong effects on BMI were also noted for the fat-to-lean mass ratio, cardiorespiratory endurance, UBSE, and AMSE in prepubescent boys, and for the fat-to-lean mass ratio and cardiorespiratory endurance in adolescent boys. In girls, standard scores also decreased across age and BMI category groups from the UW group to the OB group in fat-to-lean mass ratio, cardiorespiratory endurance, and HR-PFI, with negative sign results in the OW and OB groups and positive sign results in the UW and NW groups. These results were confirmed by linear regression analysis showing that BMI was strongly associated with fat-to-lean mass ratio and cardiorespiratory endurance in both groups of girls and additionally with UBSE in prepubescent girls.

Our findings corroborated with those of several studies suggesting that physical fitness may play a leading role in the prevention of obesity in children and adolescents (20, 46–48). Nevertheless, our results differ from those of Chen et al. (20) and Xu et al. (47) in the curved relationship between fitness and BMI observed by these authors, suggesting that the relationships between BMI and fitness among college students are nonlinear. Underweight, overweight, and obese students performed worse on the fitness index than normal-weight students. The absence of underweight or severely underweight students in our population may explain the physical superiority of the underweight group over the normal-weight group in our study. Prospective, longitudinal cohort studies are needed in the future to better identify causal relationships and potential mechanisms.

Our results also showed that BMI was positively associated with the fat-to-lean mass ratio. As such, body composition indices commonly used by researchers have been shown to have a high discriminatory capacity to demonstrate this relationship with obesity. However, these indicators have some shortcomings as they only focus on the effect of fat and non-fat component independently and do not consider the antagonistic effects of fat mass and lean mass on the health of participants. Relevant studies have recognized the role of the fat-to-lean mass ratio in predicting health conditions in adults, such as the prediction of metabolic syndrome in adults (49). In a recent epidemiological study, Dai et al. (14) found that for each 1 standard deviation increase in the fat-to-lean mass ratio, the odds ratio for the risk of developing the nonalcoholic fatty liver disease was 1.55 vs. 1.33 in non-obese and obese males, and 1.42 vs. 1.29 in non-obese and obese females, respectively. These authors also noted that mediation analysis showed that insulin resistance, triglycerides, high-density lipoprotein cholesterol, white blood cells, and total cholesterol mediated the association of the fat-to-lean mass ratio with the risk of nonalcoholic fatty liver disease in the obese male and female subgroups (14).

Another interesting but expected finding from this study was the higher negative association between BMI and cardiorespiratory endurance, UBSE, and flexibility. Ding and Jiang (50) carried out a study on 3,066 students (2,186 males and 880 females) using a random-intercept panel model to identify between- and within-person variations. Results revealed that explosive power and cardiorespiratory endurance were negatively related to BMI regardless of gender. In contrast to our findings, a positive correlation was noticed by these authors between flexibility and within-person BMI in males and females.

Relevant studies further suggest that low levels of PF could plausibly be identified as a “red flag” (51). Low physical fitness in young people should be carefully monitored as it could affect their maturation and subsequent physical development (52). In particular, low cardiorespiratory endurance increases the risk of developing metabolic syndrome (49), cardiovascular diseases (53), and type 2 diabetes (54). Poor cardiovascular endurance also increases all-cause mortality rates even in healthy individuals and patients (55). To counteract this effect, the American College of Sports Medicine et al. (10) recommendations indicated that children aged 5–12 years should engage in at least 60 min of moderate-to-vigorous physical activity every day. This period should include a variety of aerobic activities as well as exercises that strengthen muscles and bones. Indeed, higher levels of PF in childhood, particularly cardiorespiratory fitness, muscle strength, and motor skills, have been associated with a healthier cardiovascular profile later in life (56). Optimal PF levels may mitigate metabolic breakdown associated with excess fat (11) and have positive effects on mental and skeletal health (12). For these reasons, the assessment of PF in children provides relevant clinical and public health information that can be used to maintain and improve children's health (55).

### Strengths and limitations

In contrast with previous research examining the association between BMI and health-related physical fitness (20, 25, 47), to our knowledge, this is the first study combining all five uses of health-related components, including body composition, as part of overall fitness. In addition, the present study is the first to examine the impact of the fat-to-lean mass ratio on BMI in schoolchildren aged 10–17 years. The large research sample represents all students aged 10–17 in Al-Ahsa public schools, including many girls from an area known for its conservative customs and traditions. Another strength of the study is data organized by age, gender, and adjusted BMI.

However, this research has certain limitations. First, due to the small sample size of girls, the results should be interpreted with caution and may not generalize to other Saudi girls aged 10–17. Second, boys and girls were stratified by chronological age without accounting for inter-individual differences that might exist in maturation. Therefore, the inclusion of pubertal status would be useful to properly classify the growth patterns of children with normal pubertal variants. In addition, obese participants were divided into a single group regardless of the degree of obesity. To address this issue, any participant with a BMI >40 kg/m^2^ was excluded. However, research into the degree of obesity also needs to be conducted due to the lack of studies examining differences in body composition and fitness or intervention effects for ranking the degree of obesity (55). Third, only the relationship between health-related fitness components and BMI was examined in this study. However, it is likely that other factors related to eating habits, snacking habits, sleeping habits, and physical activity also explain existing fitness levels in adolescents and children. Further studies are necessary to confirm our findings. Finally, unlike boys, physical education classes have only recently been introduced into the female education system and have not yet spread to all schools. This development can be beneficial for children for whom physical education classes are a regular part of their weekly routine. Therefore, the absolute PF values reported in this study should be interpreted with caution.

## Conclusions

Referring to the BMI percentile charts by age and gender, the overall prevalence of overweight and obesity among students aged 10–17 years in public schools in the Al-Ahsa governorate was 10.4 and 24.7% for boys and 10 and 8.4% for girls, respectively. The HR-PFI indicated that fitness levels did not differ at all between boys and girls. However, when related to sex and age category, significant differences between the boys' and girls' groups were noted in cardiorespiratory endurance, UBSE, and AMSE. Stratified by BMI category, overall fitness index and its components in boys, and fat-to-lean mass ratio, cardiorespiratory endurance, and HR-PFI in girls showed significant differences between groups with decreasing values in the order of UW, NW, OW, and OB groups. Multiple linear regressions showed that BMI was positively associated with the ratio of fat-to-lean body mass and negatively associated with cardiorespiratory endurance in the total group of participants as well as the subgroups by sex and age categories. BMI was also negatively associated with flexibility and HR-PFI in the total group, UBSE, AMSE, and HR-PFI in prepubescent boys, and UBSE in prepubescent girls. The coefficient of determination values was 0.65 in the total group, 0.72 in prepubescent boys, 0.863 in adolescent boys, 0.956 in prepubescent girls, and 0.818 in adolescent girls.

## Data availability statement

The raw data supporting the conclusions of this article will be made available by the authors, without undue reservation.

## Ethics statement

The studies involving human participants were reviewed and approved by Ethical Committee of the Deanship of Scientific Research, King Faisal University (Ref. No. KFU-REC-2021-OCT-EA00019). Written informed consent to participate in this study was provided by the participants' legal guardian/next of kin.

## Author contributions

MAS, MMA, and MSA were the project managers and conceived the manuscript designs and analyzed the literature. IA, KA-S, and MA participated in the design of the manuscript and collected data. MAS drafted the manuscript. MMA, IA, KA-S, MA, and MSA revised the content of the manuscript. MAS and MSA are the guarantors of this work and, as such, had full access to all the data in the study and take full responsibility for the integrity of the data and the accuracy of the data analysis. All authors contributed to the article and approved the submitted version.

## Funding

The Deanship of Scientific Research, King Faisal University, Al-Ahsa 31982, Saudi Arabia, financed this study (GRANT1171).

## Conflict of interest

The authors declare that the research was conducted in the absence of any commercial or financial relationships that could be construed as a potential conflict of interest.

## Publisher's note

All claims expressed in this article are solely those of the authors and do not necessarily represent those of their affiliated organizations, or those of the publisher, the editors and the reviewers. Any product that may be evaluated in this article, or claim that may be made by its manufacturer, is not guaranteed or endorsed by the publisher.
